# Comparison of methods for quick estimation of psychometric thresholds

**DOI:** 10.3389/fnins.2026.1760278

**Published:** 2026-07-22

**Authors:** Adrien Chopin, Martin Szinte, Angelo Arleo, Denis Sheynikhovich

**Affiliations:** 1 Smith-Kettlewell Eye Research Institute, San Francisco, CA, United States; 2 Sorbonne Université, INSERM, CNRS, Institut de la Vision, Paris, France; 3 Institut de Neurosciences de la Timone, CNRS, Aix-Marseille Université, Marseille, France

**Keywords:** adaptive estimation, Bayesian method, perception, psychometrics, psychophysics

## Abstract

Threshold estimation plays a crucial role in modern cognitive science. Numerous psychophysical methods exist to quickly estimate key psychometric function parameters, like thresholds. Using Monte Carlo simulations of 60 to 120 trials, we compare stimulus selection methods in their ability to estimate thresholds. We evaluate model performance using measures including parameter bias, interquartile range, half-width of the limits of agreement and probability of missing the detection of participants responding randomly (miss rate). Psychometric functions are classically assumed to be monotonic, but they can be non-monotonic, with performance peaking and decreasing. Therefore, we investigate methods estimating threshold parameters for non-monotonic participants. We also assess methods' ability to accurately identify ground-truth threshold parameters when participants respond randomly, or have parameters outside the normal range. For monotonic participants, Bayesian methods outperform the Method Of Constant Stimuli (MOCS) by an order of magnitude. For non-monotonic participants, when methods assume monotonicity, even using 120 trials results in catastrophic inaccuracy with median biases over 4,000%. When assuming non-monotonicity instead, methods result in psychometric properties comparable to those obtained for monotonic participants. However, MOCS, Psi and psi-grid inaccurately identify participants responding randomly, showing miss rates over 21%. Two methods in the Psi family outperform other methods, reaching results comparable to the monotonic case. In all tested scenarios, the best methods are psi-marginal and psi-marg-grid, a new method that combines psi-marginal and psi-grid. When these two methods assume non-monotonicity, they impose little performance cost when evaluating truly monotonic functions (example of cost: miss rate increased from 0 to 4% for identifying participants responding randomly with 60 trials) and no cost with 120 trials. Among these methods, we therefore recommend psi-marg-grid and psi-marginal for efficient threshold estimation regardless of potential monotonicity.

## Introduction

Psychophysical methods are used to characterize human perception. They allow the experimenter to extract important parameters of a participant's psychometric function (the function linking stimulus intensity to perceptual performance). The most important parameter extracted is the threshold, which is the stimulus intensity leading the participant to be correct with a given probability (often 75%). Numerous studies compared psychophysical methods in their ability to estimate participants' thresholds accurately and precisely (Leek, 2001; King-Smith et al., 1994; Remus and Collins, 2008; Shen et al., 2015; García-Pérez and Alcalá-Quintana, 2005). Recent sophisticated Bayesian adaptive methods (Owen et al., 2021; Keeley et al., 2023) have been developed for multidimensional parameter estimation. However, a sizable fraction of the clinical and perception science community still relies on standard up-down staircases (Levitt, 1971), the Method of Constant Stimuli (MOCS) or ZEST. To the best of our knowledge, only one study (Serrano-Pedraza et al., 2020) directly compared recent evolutions of adaptive methods such as psi-marginal (Prins, 2013) and psi-grid (Doire et al., 2017) to standard sampling methods like MOCS or ZEST. To the best of our knowledge, no study specifically compares the methods' ability to detect participants who are responding randomly.

In addition, almost all studies assume that psychometric functions monotonically increase over the tested stimulus intensities. This assumption frequently fails (García-Pérez, 2014). Non-monotonic profiles occur for example in temporal generalization tasks, binary synchrony judgment tasks, specific forms of the same-different tasks, oddball tasks and ternary response tasks. Common examples include stereovision tasks (Kane et al., 2014; Ding and Levi, 2021), motion speed tasks, speech perception (Leek, 2001), pitch perception tasks (Wheatley, 2018; Bonnard et al., 2013), perimetry tasks (Turpin et al., 2010) or tasks measuring eccentricity effects on other discrimination dimensions (Poirier and Gurnsey, 2005). Researchers often discard non-monotonic data. For example, studies discarded non-monotonic data found in 11 to 55% of simulated perimetry tasks (Turpin et al., 2010) and 15% of pitch perception tasks on people with cochlear implants (Bonnard et al., 2013).

**Figure 1:**
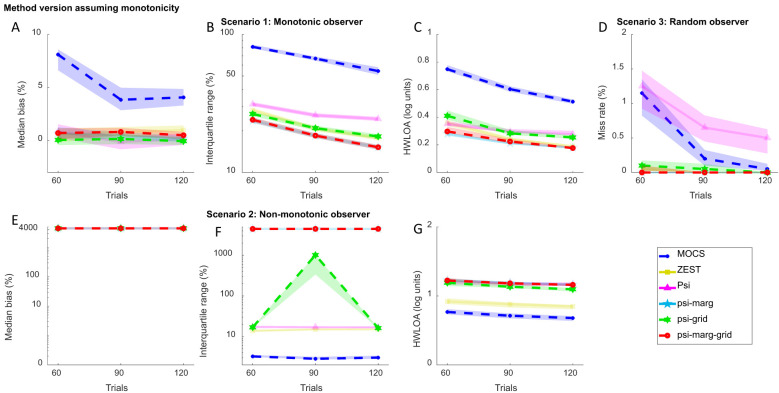
Effect of the number of trials on method versions assuming monotonicity. Simulation results comparing methods for measuring thresholds of an observer with a monotonic profile (scenario 1: **A–C**) or a non-monotonic profile (scenario 2: **E–G**). **(D)** illustrates the results for scenario 3 (test miss rates for a participant responding randomly). Performance statistics are median bias (a measure of accuracy, panels a and e), interquartile ranges (a measure of precision, **(B and F)** and the half-width of limits of agreement (HWLOA, a measure of repeatability, **C and G**), aggregating across all parameter variations. Methods are MOCS (blue dashed line with dot symbol), ZEST (yellow line with square symbol), Psi (magenta line with triangle symbol), psi-marg (cyan line with star symbol), psi-grid (green dashed line with pentagram symbol) and psi-marg-grid (red dashed line with open circle symbol). Shaded areas represent confidence intervals using interquartile ranges.

Stereovision tasks provide a clear practical illustration. Stereovision is the ability to perceive depth from binocular disparities. Observers have a minimal perceivable disparity (threshold) and a maximal perceivable disparity (upper disparity limit). Measuring stereo-deficiencies across a large disparity range risks including the upper disparity limit. Some observers show upper disparity limits as small as 480 arcsec (Kane et al., 2014), (Figure 1, right pane) and the average observer is predicted to show such low upper disparity limits under certain circumstances [for example in (Ding and Levi 2021), Figure 1, panels B, 6 cpd, monocular contrasts at 0.24 and 0.06]. This upper limit presence creates non-monotonic psychometric functions. We define non-monotonic functions as any function decreasing after reaching a maximum within the simulated threshold range.

Non-monotonicity poses a fundamental problem for many psychometric estimation methods. To converge, standard staircase methods require that “the expected proportion of positive responses is a monotonic function of stimulus level (at least over the region in which observations are made)” (Levitt, 1971). It is easy to understand why: a staircase designed to converge on a 75% correct proportion may encounter two such points in a non-monotonic function. Sampling on the descending part of the function causes incorrect responses to increase the stimulus level, breaking the converging mechanism. Adaptive Bayesian methods targeting specific percent-correct points (e.g., the sweet point) likely suffer similar failures given that they lack robustness to violations of their parametric assumptions (García-Pérez, 2014; Alcalá-Quintana and García-Pérez, 2004). For robust adaptive Bayesian methods not targeting a specific point, like Psi or ZEST, the effect remains unclear.

Here, we used Monte Carlo simulations to compare threshold estimation efficiency using a measure of accuracy (bias), precision (interquartile range), repeatability (half-width of the limits of agreement) and ability to flag participants responding randomly (miss rates). We evaluate several adaptive Bayesian methods against a non-adaptive MOCS benchmark. MOCS is usually inefficient, but unbiased and might become advantageous in the case of non-monotonic functions, given enough trials. The Bayesian methods include Zippy Estimation of Sequential Threshold (King-Smith et al., 1994; abbreviated ZEST), Psi (Kontsevich and Tyler, 1999), psi-marginal (Prins, 2013; abbreviated psi-marg), psi-grid (Doire et al., 2017), and a novel psi-marg-grid method (eRDS v6; Denkinger et al., 2023). Psi uses entropy for optimal stimulus placement when estimating multiple psychometric parameters simultaneously. Psi-marginal marginalizes posterior distributions on nuisance parameters. Psi-grid uses an adaptive search grid. Psi-marg-grid combines marginalization with an adaptive search grid.

All methods use identical ground-truth likelihood functions. We compare how efficiently each method samples the stimulus space to converge on an accurate threshold estimate. We study the method's robustness to a mismatch between the simulated participant profile (monotonic, non-monotonic or random) and the method assumption (monotonic or non-monotonic). We determine the method's ability to flag random responders and the effect of the number of trials.

While recent work has addressed high-dimensional spaces, our core contribution specifically focuses on low-trial-count univariate (threshold) estimation of monotonic, non-monotonic and random profiles, to improve clinical translation.

## Results

We investigated how the different methods compared to each other and how the number of trials used influenced the performance for estimating the threshold of the psychometric function. A larger number of trials generally improved the performance but rarely influenced the ranking between methods.

### Versions assuming monotonicity

Figure 1 illustrates the effect of the number of trials when the version of the method only assumes monotonicity in the observer's profile. Smaller numbers on the ordinate of each panel indicate better performance.

#### Scenario 1–monotonic profile

With the exception of MOCS, all methods yielded excellent median biases below 1%, interquartile ranges below 35%, and half-widths of the limits of agreement (HWLOA) below 0.4 log-units. MOCS performed significantly worse in terms of accuracy (biases >3.5%), precision (interquartile ranges >50%) and repeatability (HWLOAs >0.5 log-units). Psi-marg and psi-marg-grid yielded the best precision interquartile range (Figure 1b) and mean HWLOAs (Figure 1c). The other methods yielded intermediate performances. When the number of trials increased, performance generally increased.

#### Scenario 2–non-monotonic profile

All methods seriously failed to estimate thresholds with median biases above 4,000% (Figure 1e). When considering such large biases, interquartile range and HWLOAs are not interpretable as many of the obtained thresholds are exceeding the stereoblindness criterion (Chopin et al., 2019) and will be capped (Figure 1f). The number of trials had minor effects on the performance, confirming the lack of interpretability.

#### Scenario 3–participant responding randomly

For a participant responding randomly, all methods produced miss rates below 1.1% (Figure 1d) with most of the methods (except for MOCS with 60 trials and Psi) close to or at 0%. The miss rate is the probability that a method would yield a stereoacuity better than stereoblindness (1,300”) when simulating a participant responding completely randomly. The lower the miss rate, the better the method is able to identify individuals with severe perceptual deficiencies leading to random responses.

### Versions assuming non-monotonicity

**Figure 2:**
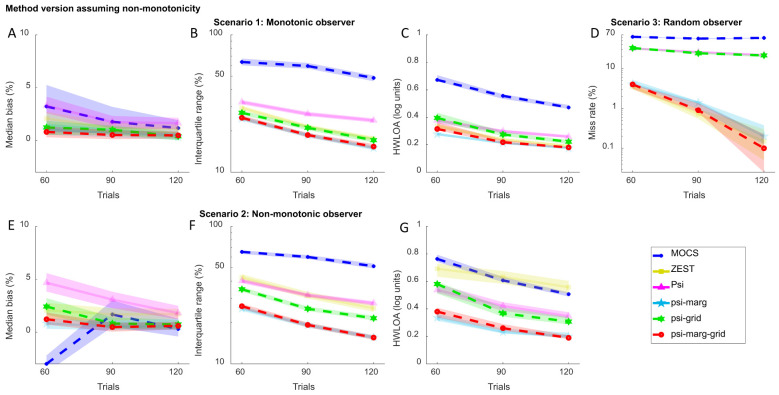
Effect of the number of trials on method versions assuming non-monotonicity. Simulation results comparing methods for measuring thresholds of an observer with a monotonic profile (scenario 1: **A–C**) or a non-monotonic profile (scenario 2: **E–G**). **(D)** illustrates the results for scenario 3 (test miss rates for a participant responding randomly). Performance statistics are median bias **(A and E)**, interquartile range **(B and F)** and HWLOA **(C and G)**, aggregating across all parameter variations. Methods are MOCS (blue dashed line with dot symbol), ZEST (yellow line with square symbol), Psi (magenta line with triangle symbol), psi-marg (cyan line with star symbol), psi-grid (green dashed line with pentagram symbol) and psi-marg-grid (red dashed line with open circle symbol). Shaded areas represent confidence intervals using interquartile ranges.

Figure 2 illustrates the effect of the number of trials when the version of the method assumes non-monotonicity in the observer's profile. Again, the number of trials improved performance but did not affect much the ordering between the methods. For all methods, larger number of trials led to an improvement in at least two of the statistics.

#### Scenario 1–monotonic profile

For observers with a monotonic profile, all methods produced a median bias below 5% and all methods with the exception of MOCS led to interquartile ranges below 35% and HWLOAs below 0.4 log units. Psi-marg-grid and psi-marg still led to the smallest median biases (Figure 2a), interquartile ranges (Figure 2b) and HWLOAs (Figure 2c), followed closely by ZEST and psi-grid.

When the observer showed a monotonic profile, the order of magnitude of each performance metric in scenario 2 was comparable to scenario 1, sometimes slightly better, sometimes slightly worse, with the exception of MOCS (Figures 2e–g). For example, several methods showed a slightly worse bias, with ZEST and Psi yielding biases above 1%. MOCS actually increased performance when assuming non-monotonicity, producing better biases, interquartile ranges and HWLOAs, despite still demonstrating the worse performance across methods. For psi-marg and psi-marg-grid at 120 trials, biases were below 0.5%, interquartile ranges ~15% and HWLOAs ~0.18 when estimating a monotonic profile, and this was true both when assuming monotonicity or not. In other words, with these two methods, there was no cost in assuming non-monotonicity with a monotonic observer at 120 trials.

#### Scenario 2–non-monotonic profile

For an observer with a non-monotonic profile, results were greatly improved in comparison to the method version assuming only monotonicity. All methods produced a median bias below 5%, and all methods but MOCS led to interquartile ranges below 42% and as good as 15.5% (psi-marg-grid and psi-marg with 120 trials). All methods reached at least a HWLOA of 0.8 and as good as 0.18 (psi-marg-grid and psi-marg with 120 trials). The best methods for bias, interquartile range and HWLOAs were again psi-marg-grid and psi-marg and the worst were ZEST, Psi and MOCS. The differences in performance between these two methods and the rest of the methods were sizeable for interquartile ranges and HWLOAs. For example, at 60 trials, psi-marg and psi-marg-grid reached interquartile ranges between 24.3 and 26.3% (vs. 34.9% for the next best: psi-grid) and HWLOAs between 0.31 and 0.38 (vs. 0.54 for the next best: Psi). The number of trials improved performance for most methods, in particular interquartile ranges: for example, psi-marg-grid led to an interquartile range of 26.3% with 60 trials and only 15.5% with 120 trials. It took about twice the number of trials to reach the same interquartile range with ZEST or Psi than with psi-marg-grid and twice the number of trials to reach the same HWLOA with Psi than with psi-marg.

#### Scenario 3–participant responding randomly

For a participant responding randomly, the methods clustered in three groups (Figure 2d). MOCS consistently obtained high miss rates (between 56.4% at 90 trials and 62.5% at 60 trials). High miss rates indicate that the method often results in measurable thresholds despite the observer responding randomly. This could partially explain MOCS' large interquartile range and HWLOA results. Psi and psi-grid obtained intermediate miss rates (between 21.4% at 120 trials and 32.7% at 60 trials for psi-grid). The other methods all obtained low miss rates, improving by an order of magnitude for each additional 30 trials: ~4% at 60 trials, ~1% at 90 trials, and ~0.15% at 120 trials. In other words, there was no cost assuming non-monotonicity with 120 trials for psi-marg, ZEST and psi-marg-grid but miss rates were slightly increased at 90 trials and significantly increased with 60 trials.

## Discussion

In the classic case of a monotonic participant tested with a psychophysical method assuming a monotonic psychometric function, we found that the methods in the Psi family were vastly superior to the method of constant stimuli at estimating thresholds, on all indicators. Two methods (psi-marg and psi-marg-grid) consistently outperformed the other methods. For example, psi-marg-grid and psi-marg were 3.6 times more precise than MOCS with 120 trials and 1.6 more precise than Psi. Psi-marg and psi-marg-grid were 11 times more accurate and 2.5 times more reliable than MOCS with 60 trials. Psi-marg-grid and psi-marg obtained similar interquartile ranges and HWLOAs with 60 trials than Psi with 120 trials.

We note that less bias with MOCS would have been obtained by fitting with a varying lapse parameter (Wichmann and Hill, 2001). Indeed, the lapse was fixed at a small non-zero value (1.75%), while simulated lapses varied from 0.5 to 3.5%. Wichmann and Hill (Wichmann and Hill, 2001) investigated the effect of using a small fixed lapse value as we did, and concluded that there is no bias as long as actual lapse values do not differ from the fixed lapse value by more than one point. This means that MOCS was unbiased in our experiment for most of the possible simulated lapse range, from 0.75 to 2.75%, but was biased for a small fraction of that range. Therefore, we note that less bias with MOCS would have been obtained if we had fit psychometric functions with a varying lapse parameter. However, this would have given an unfair advantage to MOCS because each method had the number of estimated parameters limited to two (monotonic version) or three (non-monotonic version). The exception is ZEST for which we limited the number of estimated parameters to one (monotonic version) and two (non-monotonic version). We do not think that this is giving an unfair advantage to the other methods for the following reason: Serrano-Pedraza and colleagues (Serrano-Pedraza et al., 2020) demonstrated that ZEST with an optimal slope choice is better than ZEST 2D. We now determine how optimal was the chosen slope parameter. Serrano-Pedraza and colleagues (Serrano-Pedraza et al., 2020) measured the average slope values for a group of 71 participants to be 1. It is also known that overestimating the slope of the participant's psychometric function reduces threshold bias in Bayesian methods like ZEST (Serrano-Pedraza et al., 2020; Alcalá-Quintana and García-Pérez, 2004). Therefore, we used a slope value of 2, that we think is optimal. Note that we also attempted simulations with a slope of 1 and that less bias is produced with a slope of 2.

Note also that a full evaluation of the behavioral validity of the psychometric models used here lies outside the scope of this work. The interested reader is referred to the original validations of each method (Keeley et al., 2023; Kontsevich and Tyler, 1999) and to the broader literature on psychometric function modeling (Denkinger et al., 2023).

We also found that MOCS and the tested Bayesian methods cannot be used meaningfully to estimate thresholds of participants with non-monotonic profiles. When assuming non-monotonicity, large differences in accuracy, precision, test-retest and miss rates exist between the methods, with psi-marg-grid and psi-marg consistently producing the best results. In that case, MOCS suffers from low precision, test-retest and high miss rates. While ZEST, Psi and Psi-grid can achieve some reasonable performance, there is always at least one metric that shows problematic outcomes. Indeed, Psi and psi-grid show very high miss rates (failing 21%−34% of the time to identify participants responding randomly) and ZEST shows poor test-retest (HWLOA>0.56 log units).

The number of trials does not affect these conclusions but we only tested between 60 and 120 trials. In this work, we studied non-monotonicity as simply present or absent. Future work could investigate how specific non-linearities differently impact various sampling methods.

Tasks for which these results are the most interesting are estimation of threshold in stereovision tasks (Kane et al., 2014), motion speed tasks, pitch tasks in audition (Wheatley, 2018; Bonnard et al., 2013), perimetry tasks (Turpin et al., 2010) and tasks involving eccentricity effects on other discriminations (Poirier and Gurnsey, 2005). Threshold estimation plays a key role in the cognitive science of perception. Being able to quickly and accurately estimate psychometric function parameters allows an important perceptual measure to translate into the clinic, and it opens the doors to demonstrating treatment efficiency at improving a perceptual function. Indeed, when qCSF appeared (Lesmes et al., 2006, 2010) as a way to quickly measure the contrast sensitivity function (CSF), it was quickly adopted in clinical settings (Alahmadi et al., 2018; Li et al., 2021). It is worth mentioning qCSF because the method relies on an algorithm similar to Psi, but with a strong initial prior based on normative data, which is appropriate if all participants show the same profile. This is not true when measuring stereoacuity for example, given the ratio between monotonic and non-monotonic profiles is not known. qCSF has been applied to disparity detection (Reynaud et al., 2015) assuming that disparity sensitivity follows the same dependence on spatial frequency as contrast sensitivity when presented in global random-dot stereogram corrugations. Again, the method relies on a large normative dataset. In contrast, the Bayesian methods tested in this work all used a uniform prior.

For several other tasks, the adaptive inward-outward staircase (García-Pérez, 2014) may be the best option. Indeed, the staircase is most appropriate for tasks where the parameter of interest is the peak of the non-monotonic profile: for example, temporal generalization tasks, binary synchrony judgment tasks, other forms of the same-different tasks, oddball tasks and ternary response tasks.

Recent work has extended Bayesian adaptive psychophysics beyond the univariate parametric setting. Owen and colleagues (Owen et al., 2021) introduced non-parametric GP-based adaptive sampling (AEPsych) in multidimensional spaces; Keeley and colleagues (Keeley et al., 2023) proposed a semi-parametric GP model for high-dimensional discrimination tasks. Both are complementary to the present work, which focuses on low-trial-count univariate estimation under non-monotonic psychometric functions, a regime directly relevant to clinical assessments.

Given that comparable or better results were found for participants with monotonic profiles when using methods assuming non-monotonicity, there is no or little cost in always using methods assuming non-monotonicity, as long as enough trials are used. This is particularly true for psi-marg and psi-marg-grid methods, where no cost exists at 120 trials. These two methods also show the best performance in all scenarios (ignoring scenario 2 for versions assuming monotonicity). Therefore, we recommend using psi-marg-grid or psi-marg, even in cases when the probability of finding a non-monotonic profile is not known. We recommend psi-marg when the range of stimulus intensities containing the threshold is known, because this method is simpler to implement, and psi-marg-grid when there is uncertainty about that range (for example, in pathology). When not using the versions assuming non-monotonicity, extra care should be taken to make sure that only the monotonic part of the psychometric function is represented in the range of the presented stimulus intensities.

## Methods

### Monte Carlo simulations

We simulated the following **experiment**: ideal observers performed a detection experiment, in which different intensities of stimuli were presented and participants answered which one of two stimuli contained the signal of interest. Stimulus intensities could be presented simultaneously (two-alternative forced choice−2AFC) or one after the other (two-interval forced choice−2IFC).

**Figure 3:**
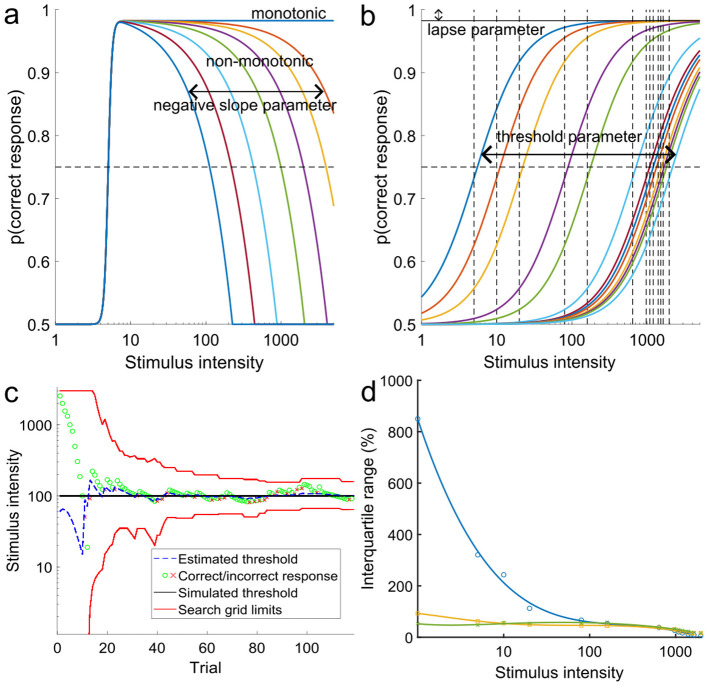
**(a)** Parametrization of the psychometric functions used for simulations. This panel illustrates the effect of changing the negative slope parameter (each color corresponds to a different parameter value). The horizontal dashed line corresponds to the 75%-correct level. The function has its threshold at 5” here and non-monotonic profiles decrease back to the 50%-correct level. The monotonic profile corresponds to the top dark-blue curve. **(b)** This panel illustrates the effect of changing the threshold parameter (each color corresponds to a different parameter value). Each dashed vertical line indicates the threshold value. More thresholds are simulated around the transition from stereo-deficient to ecological stereoblindness (Chopin et al., 2019). **a** shows functions with a positive slope parameter at 0.2 while **b** shows a value at 2.4. The lapse parameter value is illustrated on **b**. **(c)** Illustration of the adaptive search grid used in the psi-grid and psi-marg-grid methods (the depicted one is psi-marg-grid) for one particular simulation. Similar illustration for other methods can be found in Supplementary Figures S2–S6. Symbols show the intensity presented at each trial (green circle: correct response, red cross: incorrect response). The first twelve trials are trials with fixed intensities. The horizontal black line corresponds to the simulated threshold, the dashed blue line shows the evolution of the estimated threshold and the red lines show the lower and upper limits of the search grid, alternating contractions and relaxations. **(d)** Illustration of the polynomial fit used when aggregating the simulations performance metrics across simulated thresholds (x axis: stimulus intensities). The goal of this procedure was to avoid over-weighting the particular chosen thresholds used for the simulations, which were not equally spaced on the range of simulated thresholds, rendering the aggregate independent of the chosen simulated thresholds. The blue line fits 60-trials simulations (blue circles), the yellow line fits 90-trials simulations (yellow squares) and the green line fits 120-trials simulations (green crosses). These panels show parameter subsets for illustration: the complete simulated parameter values are described in the Methods.

Ideal observers followed a particular psychometric function characterized by different parameters, among which we estimated the threshold, the positive slope and the negative slope (Figures 3a, b).

We simulated three scenarios: (1) observers with a monotonic psychometric profile (null negative slope), (2) observers with a non-monotonic psychometric profile (non-null negative slope), (3) observers responding completely randomly.

We estimated two versions of each method: (1) a version assuming monotonic psychometric functions, and (2) a version adapted to assume non-monotonic psychometric functions. To adapt the function to non-monotonicity, all methods used the same psychometric (likelihood) function tailored for estimating potentially non-monotonic functions (Ψ_*NM*_) and attempted to estimate the negative slope *k* for fitting the non-monotonic part of the curve.

We tested six different methods: (1) the method of constant stimuli (MOCS), (2) ZEST (King-Smith et al., 1994), (3) Psi (Kontsevich and Tyler, 1999), (4) psi-marginal (10-abbreviated psi-marg), (5) psi-grid (Doire et al., 2017), and (6) a new method called psi-marg-grid (Denkinger et al., 2023), mixing psi-marginal's marginalization on nuisance parameters and psi-grid's adaptive search grid.

We simulated each estimation method, scenario and version with 2,000 repetitions for each simulated threshold. For illustration purposes, we used intensity values that were compatible with a stereoacuity experiment. Stimulus intensities were therefore disparities in arcsec (“), and the task was to decide which one of two stimuli was closer (while one was at zero disparity). For each estimation method, we also introduced a realistic situation in which an experimenter was interested in characterizing the pathological absence of the ability that they measure (i.e., blindness, here “stereoblindness”). Various degrees of stereoblindness were simulated, from observers having no stereoscopic ability (total stereoblindness, responding completely randomly) to observers having mild to strong stereo-deficiencies, with thresholds either above or below our criterion value for stereoblindness at 1,300”, a value representing ecological stereoblindness (Chopin et al., 2019).

Ideal observers were estimated with 60, 90 or 120 trials, realistic numbers of trials that can be used to assess thresholds in a short time, mitigating the risk of triggering perceptual learning or fatigue, that would change the estimated threshold. For all Bayesian methods, we used an initial flat prior, which is shown to be unbiased even with as few as 30 trials (Alcalá-Quintana and García-Pérez, 2004).

#### Psychometric function used for estimating parameters

The likelihood function used for estimating observer's psychometric function's parameters was represented by a logistic function Ψ of log-intensity (Serrano-Pedraza et al., 2016a). In addition to the monotonic form Ψ_*M*_ (Equations 1–3), we added a non-monotonic form Ψ_*NM*_, that was the same function, adapted to decrease linearly when stimulus intensity *x* is high (Equations 4–6).


$$\begin{array}{l} {\text{Ψ}}_{M}\left(x,\theta ,\sigma \right)=g+\;\frac{1-\;\lambda \;-g}{1\;+\;\exp\left(-b\;\left[a+x\;-\theta \right]\right)} \end{array}$$ (1)

$$\begin{array}{l} b=\;\frac{2}{\sigma }\ln\left(\frac{1-\;\lambda \;-g-\;\delta }{\delta }\right) \end{array}$$ (2)

$$\begin{array}{l} a=\;\frac{1}{b}\;\ln\left(\frac{1-\;\lambda \;-p}{p-g}\right) \end{array}$$ (3)

Ψ is the proportion of correct answers.

*x* is the base-10 logarithm of the presented intensity, e.g., disparities in arcsec (chosen by the method during stimulus placement phase).

*p* is the performance level defined as sensitivity threshold (fixed to 75%).

θ is the detection threshold for the stimulus, in log-intensity, i.e., the value of *x* at which Ψ reaches *p* (estimated, see Figure 3b).

λ is the lapse rate, which is the probability of error at maximum stimulus intensity (fixed to 1.75%).

*g* is the guess rate, i.e., the probability which would be correct by chance (fixed to 50%, because we simulate two-alternatives choices).

σ is the positive slope, or spread parameter, controlling how suddenly performance increases with intensity (estimated).

δ controls where we consider the top/bottom of the psychometric function (fixed to 0.01).

The parametrization has the property that θ and σ can vary independently.

The non-monotonic form is:


$$\begin{array}{l} {\text{Ψ}}_{NM}\left(x,\theta ,\sigma ,k\right)=\max\left(\left\{0.5,\;{\text{Ψ}}_{M}\left(x,\theta ,\sigma \right)c\right\}\right) \end{array}$$ (4)

$$\begin{array}{l} c=\min\left(\left\{1,\;1-\frac{k}{100}\left(10^{x}\;-\;d\;\right)\right\}\right) \end{array}$$ (5)

$$\begin{array}{l} d=\;10^{\theta }+\frac{\sigma }{2}10^{x} \end{array}$$ (6)

*k* is the negative slope parameter, controlling how rapidly performance decreases back to 50% (estimated for versions assuming non-monotonicity). When *k* = 0, Ψ_*NM*_ = Ψ_*M*_.

*d* represents the intensity at which the function starts decreasing. It is not a free parameter but is yoked to σ.

#### Ideal observer's parameter values

Simulated thresholds for ideal observers could take the values {5, 10, 20, 80, 160, 640, 970, 1,085, 1,200, 1,400, 1,515, 1,630, 1,960}, in arcsec. For each repetition, some parameters had a fixed value (same as stated above), and other parameters could take a random value. The random values of the positive slope σ were uniformly distributed between 0.2 (sudden rise) and 2 (slow rise), the negative slope between 0 (monotonic) and 0.112 (strongly non-monotonic) for non-monotonic profiles (scenario 2), or fixed to 0 for monotonic profiles (scenario 1), and the lapse uniformly distributed between 0.5 and 3.5%.

#### Method of constant stimuli (MOCS)

In the method of constant stimuli, we collected data for repetitions of pre-determined stimulus intensities, followed by an estimation of the psychometric function to extract the threshold. It was therefore the only non-adaptive method tested, and a benchmark.

##### Stimulus placement

Stimulus intensities were chosen among nine levels logarithmically spaced between 1” and 3,000” and each level was repeated for 7–13 trials, for a total of 60 to 120 trials in random order. A typical psychophysical experiment would usually have more repetitions, and levels more focused around the likely threshold, but we were interested in estimating methods capable of quickly estimating both acuity and pathology, so that this wide range was necessary.

##### Final estimate

After collecting the responses to the 90 trials, the probability of correct response was calculated for each intensity (psychometric function). Chi-square minimization was used to model the psychometric function using Ψ_*M*_ or Ψ_*NM*_, depending on the version of the method and to extract the threshold θ. When fitting the psychometric function, estimated parameters (θ, σ* and k*) were allowed to respectively vary between 0.1” and 100,000”, between 0.2 and 2.4 and between 0 and 0.224 for the non-monotonic version or simply fixed to zero for the monotonic version. To avoid dependence on starting parameters, the minimization was repeated 20 times with random starting parameters and the parameters with the lowest chi square were chosen.

#### ZEST

We implemented a 2AFC-version of the ZEST algorithm similar to the one used in the Asteroid stereotest (Serrano-Pedraza et al., 2016a,b), which is a Bayesian adaptive procedure for one parameter. Indeed, only the threshold parameter θ was estimated. The θ-space for threshold distribution was made of 348 linearly-spaced log-intensities sampled between log10(1”) and log10(3,000”). Positive slope σ was fixed to 2, negative slope at 0 for the monotonic version, and at 0.0075 for the non-monotonic version.

##### Stimulus placement

The prior distribution of θ (threshold) on the first trial was uniform. The likelihood function of θ was the logistic function Ψ_*M*_ or Ψ_*NM*_ of log-intensity, depending on the method version. For the first twelve trials, the presented intensities *x* were (in log10) {2,500”, 2,000”, 1,500”, 1,300”, 1,000”, 800”, 500”, 300”, 200”, 100”, 50”, 20”}, in that order, similar to a scaffolding practice. On the next trials, *x* was chosen as the average of the possible thresholds, weighted by the prior distribution for that trial. The posterior distribution of θ was calculated after each trial by multiplying the prior distribution by the likelihood function Ψ(*x*, θ) following a correct response at that trial and 1−Ψ(*x*, θ) following an incorrect response. The posterior distribution at trial *i* became the prior distribution at trial *i*+*1*.

##### Final estimate

The estimated threshold was the average of the possible thresholds, weighted by the last posterior distribution.

#### Psi

We implemented a one look-ahead Psi method (Kontsevich and Tyler, 1999), jointly estimating the threshold and positive slope parameters for the monotonic version, and also the negative slope parameter for the non-monotonic version. This was a Bayesian adaptive procedure using expected entropy to find the stimulus to display that would be the most informative in the parameter search space. Probability distributions were expressed in the parameter space (*x*, θ, σ, *k*). *x*, the possible next stimulus intensity, was represented by 50 values linearly spaced between log10(1”) and log10(3,000”), with the following added values (in log10): {10”, 100”, 1,000”, 2,000”, 3,000”}. θ, the threshold, was represented in the same way with 100 values, with the following added values (in log10): {10”, 100”, 1,000”, 2,000”, 100,000”}. σ, the positive slope, was represented by eight values {0.2, 0.2852, 0. 4068, 0.5801, 0.8274, 1.18, 1.6828, 2.4} and, *k*, the negative slope, by the values {0, 0.003, 0.006, 0.012, 0.024, 0.056, 0.112, 0.224} for the non-monotonic version, and fixed to zero for the monotonic version. We used a one-look-ahead search because it is as good as a two-look-ahead search (Doire et al., 2017).

##### Stimulus placement

We followed the original methods for Psi (Kontsevich and Tyler, 1999) with the following changes. We added the negative slope parameter *k* in the search space for the non-monotonic version. The prior distribution of (θ, σ, *k*), on the first trial was uniform. The likelihood function was the logistic function Ψ_*M*_(*x*, θ, σ), or Ψ_*NM*_(*x*, θ, σ, *k*), depending on the method version. For the 12 first trials, the presented intensities *x* were always {2,500”, 2,000”, 1,500”, 1,300”, 1,000”, 800”, 500”, 300”, 200”, 100”, 50”, 20”}, in that order, similar to a scaffolding practice. The expected posterior distribution was calculated at each trial by multiplying the prior distribution by the likelihood function Ψ following a correct response, and by 1−Ψ following an incorrect response, and by dividing respectively by the probability of a correct or an incorrect response. *x* was chosen to minimize the expected entropy average on the parameter space. After the response, the posterior was defined as the expected posterior distribution (θ, σ, *k*) for the presented intensity *x*, updated according to the success or failure at intensity *x*. The posterior at trial *i* became the prior at trial *i* + 1.

##### Final estimate

The estimated threshold was the average of the possible thresholds, weighted by the last posterior distribution.

#### psi-marginal (psi-marg)

We implemented a method identical to *Psi* described above, with the following changes. The positive slopes used were {0.2, 0.3, 0.4, 0.6, 0.8, 1.2, 1.6, 2.4}. Before calculating the expected entropy, the expected posterior distributions for a correct or incorrect response were marginalized over the positive and negative slope parameters (Prins, 2013). This defined the positive and negative slope parameters as nuisance parameters, and the threshold as the parameter of interest. The final estimated threshold was the average of the possible thresholds, weighted by the last marginalized posterior distribution.

#### psi-grid: psi with an adaptive search grid

We implemented a method identical to *Psi* described above, with the following changes.

#### Stimulus placement

The search grid for the threshold and positive slope parameters could be rescaled similar to a recent study (Doire et al., 2017). After each trial, except the first twelve, the mean μ and variance *v* of the updated posterior distributions were calculated according to the following Equations 7–9:


$$\begin{array}{l} u_{\theta }\left(z_{n}\right)=\;\sum_{i}\theta_{i}P\left(\theta_{i}|z_{n}\right) \end{array}$$ (7)

$$v_{\theta }(z_{n})\;=\;\sum_{i}{\left(\theta_{i}-u_{\theta }(z_{n})\right)}^{2}P\left(\theta_{i}|z_{n}\right)$$ (8)

$$\begin{array}{l} z_{n}=\;\left[x_{1},r_{1},\ldots ,x_{n},r_{n}\right] \end{array}$$ (9)

With *z*_*n*_, a vector of n observed data, pairing presented intensity *x*_1_ with response *r*_1_. There was an equivalent set of Equations 7, 8 for each estimated parameter obtained by replacing θ with the relevant parameter in the equation. The search grid limits were then calculated as $u\pm 4\sqrt{v}$ for each parameter. After the 12 first trials, if these limits matched those of the last used search grid limits within 10% when converted in linear scale, then no rescaling was performed. Otherwise, the search grid was rescaled, by resampling between $u-4\sqrt{v}$ and $u+4\sqrt{v}$, 50 logarithmically-spaced values for *x*, 100 logarithmically-spaced values for the threshold parameter and eight for the positive slope parameter (Figure 3c). In all cases, the limits were bounded between log10(1”) and log10(2,200”) (between log10(0.1”) and log10(3,000”) after the first rescaling) for *x* and θ, the values (in log10) {1”, 10”, 100”, 1,000”, 2,000”, 3,000”} were added to the *x*-space, and the values {1”, 10”, 100”, 1,000”, 2,000”, 100,000”} to the θ-space (after converting in log10). In these spaces, prior and posterior distributions were then recomputed following the entire history of presented intensities and correct/incorrect responses, trial by trial.

#### psi-marg-grid: psi-marginal with an adaptive search grid

We implemented a method identical to *psi-grid* described above, with the following changes. We only used the adaptive search grid for the threshold parameter (Figure 3c). As for *psi-marg*, before calculating the expected entropy, the expected posterior distributions for a correct or incorrect response were marginalized across the nuisance parameters (Prins, 2013): the positive and negative slope parameters.

### Analysis

For each method, version and scenario, we collected the distribution of thresholds estimated for each simulated threshold (2,000 repetitions). Note that these threshold distributions aggregate data coming from different random parameters (e.g., lapses, positive and negative slopes). Any estimated or simulated threshold >1,300” was capped at 1,300” following the concept of ecological stereoblindness (Chopin et al., 2019), and evaluated against 1,300”. To extract the performance metrics below, we aggregated across all parameter variations. To aggregate each performance metric across simulated thresholds, we first applied a polynomial fit to the performance metric expressed as a function of the simulated threshold (Figure 3d). The polynomial fit was then averaged across all thresholds between log10(5”) and log10(1,500”) with a step of 0.01 log units, to produce average performance metrics. The goal of this procedure was to avoid over-weighting the particular chosen thresholds used for the simulations, which were not equally spaced on the range of simulated thresholds. For averaging, we use the geometric mean when averaging log units (HWLOA) and arithmetic mean otherwise. The operation was repeated for each number of simulated trials (60, 90 and 120). For all performance metrics below, the closer to zero, the better the method.

#### Median bias and interquartile range

For each repetition, we calculated the difference between estimated and simulated thresholds, and for each simulated threshold, we extracted the median of the 2,000 repetitions, which we call the median bias (Doire et al., 2017; García-Pérez, 2014), and the interquartile range (75th percentile minus 25th percentile), both expressed as a percentage of the simulated threshold. The median bias is a measure of a method's accuracy while the interquartile range is a measure of its precision. We used non-parametric estimators because of the non-normal and bimodal distributions that were expected when estimating non-monotonic profiles.

#### Half-widths of limits of agreement (HWLOA)

The half-widths of limits of agreement have the goal of estimating the test-retest reliability of the methods. We randomly chose two threshold estimates among the 2,000 repetitions and calculated the difference of their logarithm (base 10), and repeated this procedure 10,000 times. The HWLOA was defined as two times the standard deviation of these differences.

#### Miss rates

Miss rates are defined as the probability of obtaining a threshold better than what is expected if the observer responds randomly (here, a stereoacuity below 1,300”, our stereoblindness criterion) when simulating an observer responding completely randomly (scenario 3). In other words, miss rates express the probability of failing to detect participants responding randomly, by attributing them a better threshold than stereoblindness.

## Data Availability

The datasets presented in this study can be found in online repositories. The names of the repository/repositories and accession number(s) can be found below: https://osf.io/uqkpd.
