# Supplementary material for: Comparison of methods for quick estimation of psychometric thresholds
Source: Front Neurosci. 2026 Jul 22;20:1760278. doi: 10.3389/fnins.2026.1760278 (PMC13439673; doi:10.3389/fnins.2026.1760278)
Supplement: Supplementary file 1 [file Data_Sheet_1.docx]

## Supplementary Material

**Supplementary figure 1:** Example of MOCS estimation for a monotonic ideal observer with threshold 50” and 90 trials (**left panel**) and 120 trials (**right pane**l): Probability of being correct as a function of stimulus intensity. The method assumes monotonicity here. Blue circles: simulated data averaged for each of the nine levels. Orange line: estimated psychometric function. Red line: final estimated threshold.

**Supplementary figure 2:** Example of ZEST estimation for a monotonic ideal observer with threshold 50” and 120 trials. The method assumes monotonicity here. Posterior distribution at the last trial (red curve) for the threshold parameter (**left panel**). The black solid line indicates the simulated parameter value and the red dashed line indicates the parameter estimate at the last trial. **Central panel**: estimated likelihood function for the final threshold (red curve) and final threshold estimate (red vertical dashed line) for presented stimuli (black circles, averaged 15 trials by 15 trials). The black curve indicates the simulated likelihood function and the solid black line indicates the simulated threshold. **Right panel**: trial-by-trial evolution of threshold estimation (dashed red curve) as a function of correct (black circles) and incorrect responses (red crosses). The solid black line indicates the simulated threshold. curve).

**Supplementary figure 3**: Example of Psi estimation for a monotonic ideal observer with threshold 50” and 120 trials. The method assumes monotonicity here. Posterior marginalized distribution at the last trial (red curve) for threshold parameter (**top left panel**) and positive slope parameter (**top central panel**): note that Psi does not involve marginalization and that marginalization here is only provided for visualization purposes. The black solid line indicates the simulated parameter value and the red dashed line indicates the parameter estimate at the last trial. On the top left panel, the probability of being stereoblind is calculated as the sum of the posterior distribution equaling or exceeding the criterion for stereoblindness. **Bottom left panel**: estimated likelihood function for the final threshold (red curve) and final threshold estimate (red vertical dashed line) for presented stimuli (black circles, averaged 24 trials by 24 trials). The black curve indicates the simulated likelihood function and the solid black line indicates the simulated threshold. **Bottom central panel**: trial-by-trial evolution of threshold estimation (dashed red curve) as a function of correct (black circles) and incorrect responses (red crosses). The solid black line indicates the simulated threshold. **Bottom right panel**: trial-by-trial evolution of positive slope estimation (dashed red curve). The solid black line indicates the simulated parameter.

**Supplementary figure 4**: Example of Psi-grid estimation for a monotonic ideal observer with threshold 50” and 120 trials. The method assumes monotonicity. Posterior marginalized distribution at the last trial (red curve) for threshold parameter (**top left panel**) and positive slope parameter (**top central panel**): note that Psi does not involve marginalization and that marginalization here is only provided for visualization purposes. The black solid line indicates the simulated parameter value and the red dashed line indicates the parameter estimate at the last trial. The red horizontal line indicates the search grid range at the last trial. The p(stereoblind) is calculated as the sum of the posterior distribution >= to the criterion for stereoblindness. **Bottom left panel**: estimated likelihood function for the final threshold (red curve) and final threshold estimate (red vertical dashed line) for presented stimuli (black circles, averaged 15 trials by 15 trials). The black curve indicates the simulated likelihood function and the solid black line indicates the simulated threshold. **Bottom central panel**: trial-by-trial evolution of threshold estimation (dashed red curve) as a function of correct (black circles) and incorrect responses (red crosses). The solid black line indicates the simulated threshold and the solid red curves the range of parameter search grid. **Bottom right panel**: trial-by-trial evolution of positive slope estimation (dashed red curve). The solid black line indicates the simulated parameter and the solid red curves the range of parameter search grid.

**Supplementary figure 5**: Example of Psi-marg estimation for a monotonic ideal observer with threshold 50” and 120 trials. The method assumes monotonicity here. Posterior marginalized distribution at the last trial (red curve) for threshold parameter (**top left panel**) and positive slope parameter (**top central panel**). The black solid line indicates the simulated parameter value and the red dashed line indicates the parameter estimate at the last trial. The p(stereoblind) is calculated as the sum of the posterior distribution >= to the criterion for stereoblindness. **Bottom left panel**: estimated likelihood function for the final threshold (red curve) and final threshold estimate (red vertical dashed line) for presented stimuli (black circles, averaged 15 trials by 15 trials). The black curve indicates the simulated likelihood function and the solid black line indicates the simulated threshold. **Bottom central panel**: trial-by-trial evolution of threshold estimation (dashed red curve) as a function of correct (black circles) and incorrect responses (red crosses). The solid black line indicates the simulated threshold. **Bottom right panel**: trial-by-trial evolution of positive slope estimation (dashed red curve). The solid black line indicates the simulated parameter.

**Supplementary figure 6**: Example of Psi-marg-grid estimation for a monotonic ideal observer with threshold 50” and 120 trials. The method assumes monotonicity here. Posterior marginalized distribution at the last trial (red curve) for threshold parameter (**top left panel**) and positive slope parameter (**top central panel**). The black solid line indicates the simulated parameter value and the red dashed line indicates the parameter estimate at the last trial. The red horizontal line indicates the search grid range at the last trial. The p(stereoblind) is calculated as the sum of the posterior distribution >= to the criterion for stereoblindness. **Bottom left panel**: estimated likelihood function for the final threshold (red curve) and final threshold estimate (red vertical dashed line) for presented stimuli (black circles, averaged 15 trials by 15 trials). The black curve indicates the simulated likelihood function and the solid black line indicates the simulated threshold. **Bottom central panel**: trial-by-trial evolution of threshold estimation (dashed red curve) as a function of correct (black circles) and incorrect responses (red crosses). The solid black line indicates the simulated threshold and the solid red curves the range of parameter search grid. **Bottom right panel**: trial-by-trial evolution of positive slope estimation (dashed red curve). The solid black line indicates the simulated parameter.
